# The Prognostic Significance of *EGFR* Adjusted Variant Allele Frequency on First-Line Osimertinib Efficacy in Advanced EGFR-Mutant NSCLC

**DOI:** 10.3390/curroncol33080439

**Published:** 2026-07-23

**Authors:** William J. Phillips, Russell Leong, Benjamin Yeung, Ahmed Al Lawati, D. Ross Camidge, Bryan Lo, Paul Wheatley-Price

**Affiliations:** 1 University of Colorado Cancer Center, Aurora, CO 80045, USA; 2 Faculty of Medicine, University of Ottawa, Ottawa, ON K1N 6N5, Canada; 3 Molecular Oncology Diagnostics Laboratory, The Ottawa Hospital, Ottawa, ON K1H 8L6, Canada; 4 Department of Medicine, University of Ottawa, Ottawa, ON K1H 8M5, Canada

**Keywords:** EGFR, non-small cell lung cancer, variant allele frequency, metastatic

## Abstract

**Introduction:** The prognostic significance of *EGFR* variant allele frequency (VAF) remains unclear in advanced *EGFR*-mutant (*EGFR*-mt) NSCLC. We examined the relationship of *EGFR* VAF and clinical outcomes in patients treated with first-line osimertinib. **Methods:** This retrospective cohort study evaluated patients with advanced *EGFR*-mt NSCLC treated at the Ottawa Hospital between July 2021 and June 2023. Baseline characteristics were collected from electronic medical records. Adjusted VAF (aVAF) was calculated by normalizing *EGFR* VAF to tumor cellularity and analyzed according to predefined thresholds: aVAF < 50% (low) and aVAF > 100% (high). The primary outcome was progression-free survival (PFS). **Results:** Of 141 patients diagnosed with EGFR-mt NSCLC, 59 were included. The median age was 70 years, and 70% were females. There were 23 (39.0%) cases with low aVAF and 16 (27.1%) with high aVAF. Neither aVAF < 50% (HR = 1.01; *p* = 0.76) or aVAF > 100% (HR = 0.81; *p* = 0.57) was associated with PFS. However, ECOG performance status ≥ 2 (vs 0–1; HR = 3.63, *p* < 0.001), EGFR exon 19 deletion (vs. L858R; HR = 0.50, *p* = 0.021), and liver involvement (HR = 2.65, *p* = 0.005) were associated with PFS on multivariable analysis. **Discussion**: *EGFR* aVAF was not associated with clinical outcomes. However, established prognostic factors including ECOG performance status, *EGFR* mutation subtype, and liver involvement were associated with PFS.

## Simple Summary

*EGFR* adjusted variant allele frequency (aVAF) is a molecular testing metric postulated to reflect how much of a tumor is made up of cells carrying the *EGFR* mutation. In this study, we examined whether *EGFR* aVAF was associated with outcomes in patients with advanced *EGFR*-mutant NSCLC treated with first-line osimertinib. We found that patients had similar progression-free survival and overall survival regardless of their tumor’s *EGFR* aVAF value, suggesting that it is unlikely to be useful for predicting outcomes, although larger studies are needed to verify these findings.

## 1. Introduction

*EGFR*-mutant (*EGFR*-mt) non-small cell lung cancer (NSCLC) makes up approximately 15–20% of cases in North America and Europe and 35% in Eastern Asia [1,2]. *EGFR* mutations are enriched in younger, female patients diagnosed with adenocarcinoma with little or no tobacco exposure [3]. Molecular testing for *EGFR* mutations can be performed using a range of methods. These include targeted assays such as a real-time polymerase chain reaction (PCR) or next-generation sequencing (NGS). NGS has been widely adopted given its declining cost and ability to simultaneously interrogate multiple molecular alterations [4,5].

Variant allele frequency (VAF) is defined as the proportion of sequencing reads at a specific genomic locus containing a specific mutant allele and is expressed as a fraction (0–1) or percentage (0–100%). For tissue-based molecular testing, the expected VAF is approximately 100% for homozygous germline variants and 50% for heterozygous germline variants. Because germline variants are present in all nucleated cells, their VAF can be interpreted without adjustment for tumor cellularity.

In contrast, the interpretation of VAF for somatic alterations depends on the proportion of tumor cells that harbor the variant. Accordingly, adjusting VAF for tumor cellularity, as estimated by a pathologist, yields adjusted VAF (aVAF), which can be used to approximate mutational clonality [6]. Higher aVAF values are generally associated with clonal alterations, whereas lower aVAF values suggest subclonality, indicating that the variant is present in only a subset of tumor cells. The significance of identifying subclonal *EGFR* mutations is that it may contribute to early resistance to EGFR-targeted therapies through the presence of tumor cell populations that are not dependent on mutant *EGFR* signaling [7,8].

Classical activating *EGFR* mutations, such as exon 19 deletions and L858R mutations, are highly predictive of response to *EGFR*-targeted therapy. The current standard of care for advanced *EGFR*-mt NSCLC is treatment with third-generation EGFR tyrosine kinase inhibitors (TKIs), including osimertinib and lazertinib [9,10]. These can be either administered as monotherapy or in combination with platinum-based chemotherapy or amivantamab based on the results of FLAURA-2 and MARIPOSA, respectively [11,12]. However, even with these effective combinations, approximately 15–30% of *EGFR*-mt tumors progress within the first year of therapy, underscoring the persistent biologic heterogeneity of this disease.

The prognostic value of *EGFR* VAF remains unclear. Prior reports suggest inferior clinical outcomes in patients with lower *EGFR* VAF, but these studies are limited by small sample size, use of earlier-generation TKIs, and inconsistent normalization for tumor cellularity [13,14,15]. In this study, we examined the relationship between *EGFR* adjusted VAF (aVAF) and clinical outcomes in patients treated with first-line osimertinib monotherapy for advanced *EGFR*-mt NSCLC.

## 2. Methods

This research project was reviewed and approved by the Ottawa Hospital Research Institution (OHRI) Research Ethics Board.

### 2.1. Study Population and Design

This was an observational retrospective study of consecutive patients diagnosed with advanced *EGFR*-mt NSCLC at the Ottawa Hospital Cancer Centre (Ottawa, ON, Canada) between 1 July 2021 and 30 June 2023. Patients were included if they were 18 years of age or older, had unresectable stage III-IV disease not amenable for definitive radiation therapy or surgery, received at least 4 weeks of osimertinib, and had *EGFR* L858R or exon 19 deletion confirmed by a clinically accredited assay. Patients were excluded if they received prior systemic therapy for advanced disease or prior *EGFR*-targeted therapy in any setting.

### 2.2. Patient Characteristics

Demographic and clinical characteristics were collected from the electronic medical records, including sex, age at diagnosis, history of tobacco exposure, Eastern Cooperative Group (ECOG) performance status, metastatic sites at diagnosis, and PD-L1 expression by immunohistochemistry reported as tumor proportion score (TPS).

NGS was performed using the Oncomine Comprehensive Assay v3, with variant annotation by the Eastern Ontario Regional Laboratory Association (EORLA). *EGFR* VAF was calculated as the proportion of reads harboring the *EGFR* mutation relative to the total reads at the corresponding genomic locus. Tumor cellularity was estimated by a clinical pathologist as the proportion of tumor cells relative to total nucleated cells within the sample.

*EGFR* VAF was adjusted for tumor cellularity (*EGFR* observed VAF ÷ tumor cellularity) and expressed as a percentage. aVAF was analyzed primarily using predefined exploratory binary cutoffs: aVAF < 50% versus ≥50% (low aVAF analysis) and aVAF > 100% versus ≤100% (high aVAF analysis). After adjustment for tumor cellularity, a clonal heterozygous driver mutation is expected to have an aVAF approximately 50% assuming a diploid genome. The aVAF < 50% threshold was therefore selected to capture variants below this cutoff to identify possible subclonal alterations. In contrast, the aVAF > 100% threshold was selected to identify tumor substantially exceeding the expected clonal value, which may suggest enrichment of the mutant allele.

### 2.3. Clinical Outcomes of Interest

Real-world progression-free survival (PFS) was the primary outcome of interest. It was defined as the time from treatment initiation to radiographic progression or death from any cause. The interval of radiographic assessments was determined at the discretion of the treating oncology team, reflecting real-world clinical practice. Radiation therapy for oligoprogressive disease was considered a progression event, even if osimertinib was continued past progression.

Overall survival (OS) was evaluated as a secondary outcome and was defined as the time from osimertinib initiation to death from any cause.

### 2.4. Statistical Analysis

Categorical variables were summarized as frequencies and percentages, and continuous variables as medians with interquartile ranges (IQRs). Baseline characteristics that were significantly different between *EGFR* aVAF categories (aVAF < 50% vs. ≥50% and aVAF ≤ 100% vs. >100%), as determined by Pearson’s chi-squared test for categorical variables and Mann–Whitney U test for continuous variables, were considered potential confounders and included in multivariable analyses.

Time-to-event outcomes were analyzed using Cox proportional hazards model regression and reported as hazard ratios (HRs) with 95% confidence intervals (95% CIs). Median PFS and OS were estimated using the Kaplan–Meier method. Median follow-up time was calculated using the reverse Kaplan–Meier method. All statistical tests were two-sided, and statistical significance was set to *p* < 0.05. Statistical analysis was performed using IBM SPSS Statistics for Mac, version 29.0 (Armonk, NY, USA).

## 3. Results

### 3.1. Baseline Characteristics

**Figure 1:**
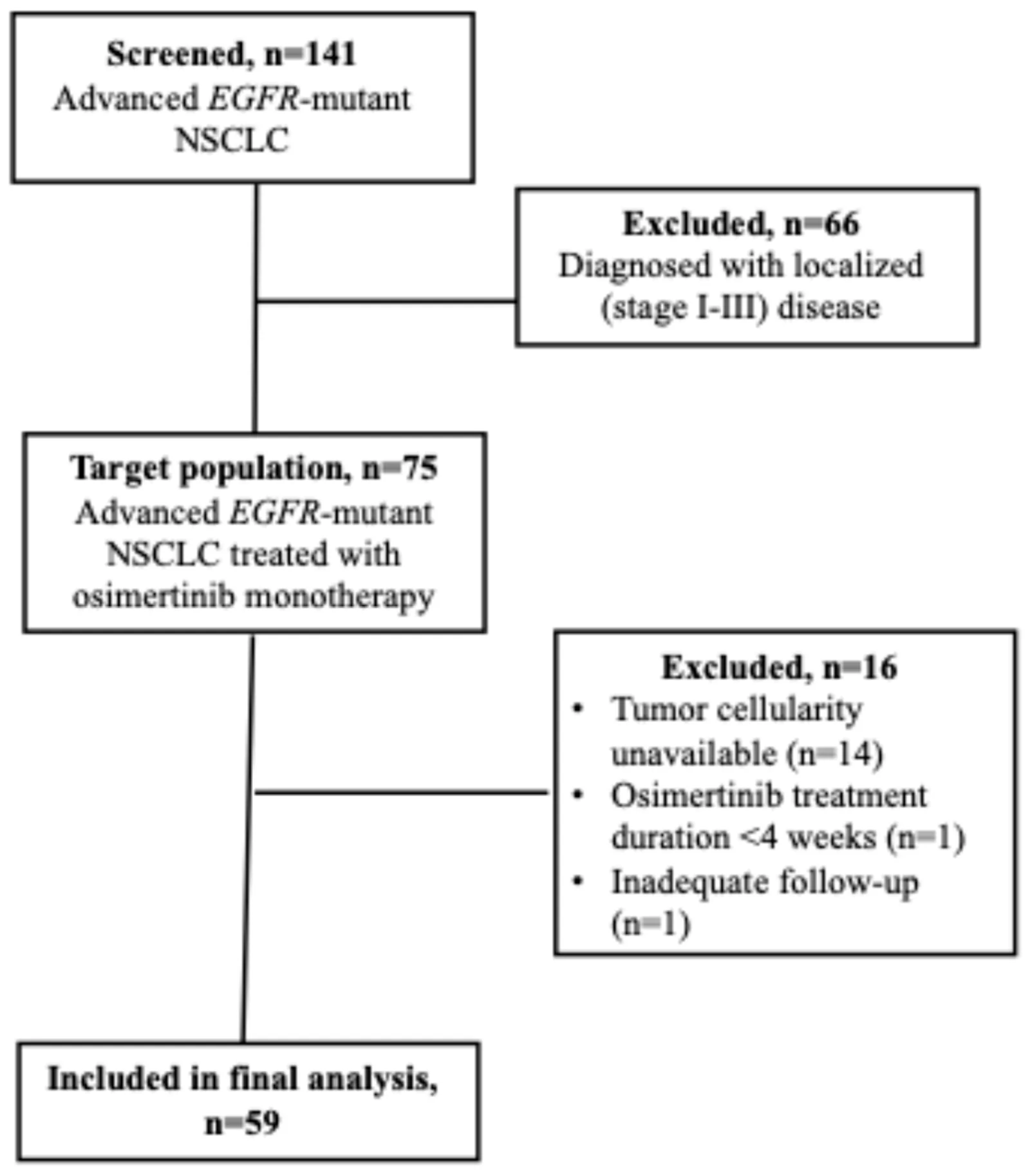
Study flow diagram outlining generation of the cohort for the primary analysis.

A total of 141 patients were diagnosed with NSCLC harboring common activating *EGFR* mutations (exon 19 deletion and L858R). Of these, 66 patients had early-stage disease and 75 had metastatic or recurrent disease treated with osimertinib monotherapy. Sixteen patients were excluded, 14 due to the absence of tumor cellularity from the pathology report, 1 who did not receive treatment with osimertinib for at least 4 weeks, and 1 who was lost to follow-up, as shown in Figure 1.

Overall, 59 patients were included in the final analysis. Baseline characteristics stratified by *EGFR* aVAF categories are summarized in Table 1. The median age was 70 years, and 70% were female. Most patients had an ECOG performance status of 0–1 (63%) and had no or light tobacco exposure (56%). *EGFR* mutation subtypes were comparable, with 30 patients (51%) with L858R mutations and 29 (49%) with exon 19 deletions. The most frequent sites of metastatic involvement were the brain (56%), bone (49%), pleura (39%), liver (27%), and adrenal gland (20%).

Associations between baseline characteristics and aVAF categories (aVAF < 50% vs. ≥50% and aVAF ≤ 100% vs. >100%) are shown in Supplementary Table S1. Pleural involvement was the only characteristic associated with aVAF < 50% (*p* = 0.027); all other variables were balanced between aVAF < 50% vs. ≥50% subgroups. No variables were significantly different between aVAF ≤ 100% vs. >100% subgroups.

### 3.2. Variant Allele Frequency

**Figure 2:**
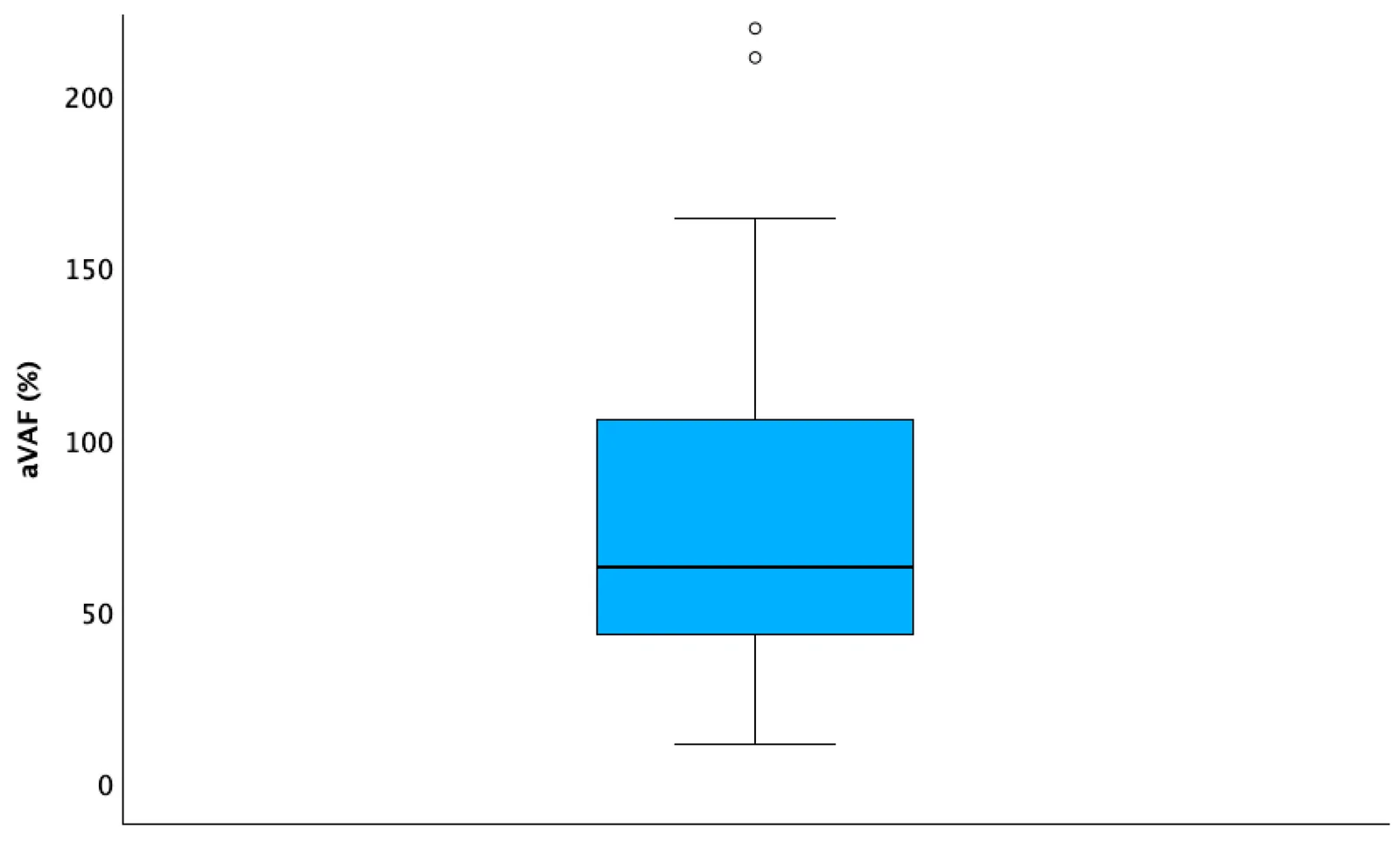
Distribution of adjusted variant allele frequency (aVAF) in the study cohort shown as a box plot. The median aVAF was 63% (IQR, 43–106). Abbreviations: aVAF, adjusted variant allele frequency; IQR, interquartile range.

The median unadjusted VAF was 35% (IQR, 18–53) and aVAF was 63% (IQR, 43–106). There were 23 (39.0%) cases with low aVAF (<50%) and 16 (27.1%) cases with high aVAF (>100%). Figure 2 depicts a box plot summarizing the distribution of aVAF.

### 3.3. Relationship of aVAF and Progression-Free Survival

At the time of analysis, the median follow-up was 40 months (95% CI = 32.2–47.8), during which 47 PFS events had occurred. The median PFS (mPFS) for the overall cohort was 19 months (95% CI = 14.8–23.3).

**Figure 3:**
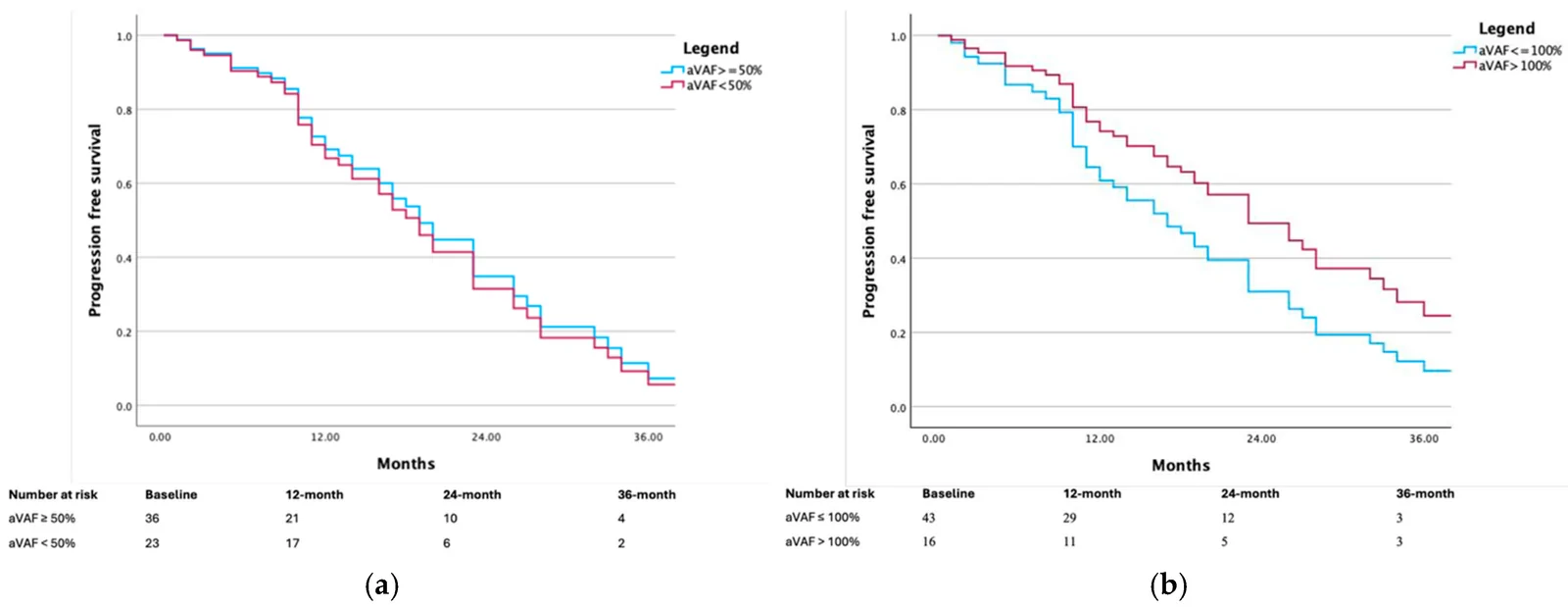
Association between adjusted variant allele frequency (aVAF) and progression-free survival using predefined thresholds: (**a**) aVAF < 50 and (**b**) aVAF > 100%. Time-to-event outcomes were analyzed and visualized using Cox proportional hazards methods. Abbreviation: aVAF, adjusted variant allele frequency.

Patients with aVAF < 50% had a mPFS of 19 months (95% CI = 15.9–22.1) compared with 16 months (95% CI = 7.6–24.4) among those with aVAF ≥ 50%. After adjusting for pleural involvement, aVAF < 50% was not associated with PFS (HR = 1.13; 95% CI = 0.62–2.08; *p* = 0.70), Figure 3a.

The mPFS was 23 months (95%CI = 7.1–38.9) in patients with aVAF > 100% and 18 months (95% CI = 14.2–21.9) among patients with aVAF ≤ 100%. aVAF > 100% was not associated with PFS (HR = 0.60; 95% CI = 0.30–1.22; *p* = 0.16), Figure 3b. There was no significant association of *EGFR* aVAF as a continuous variable with PFS (HR = 1.00, 95% CI = 0.99–1.01, *p* = 0.97).

### 3.4. Relationship of VAF and Overall Survival

Over the follow-up window, 29 OS events occurred. The median OS of the entire cohort was 30 months (95% CI = 26.7–33.3).

**Figure 4:**
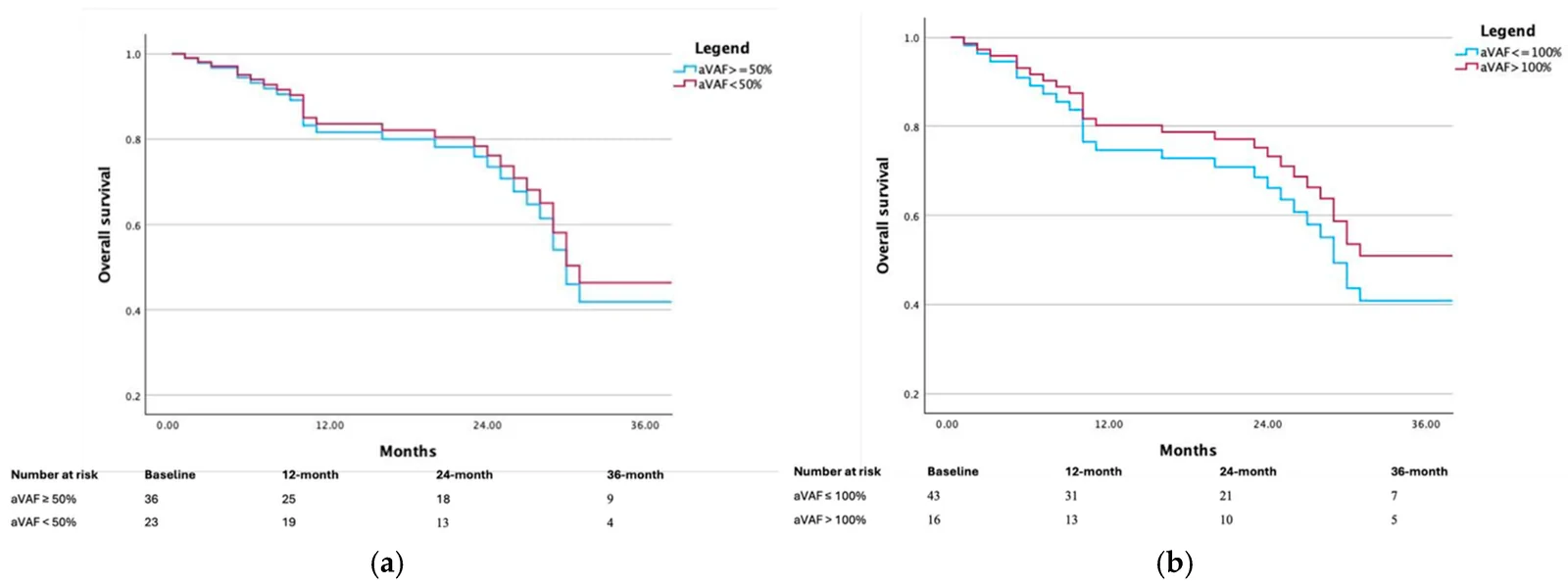
Association between adjusted variant allele frequency (aVAF) and overall survival using predefined thresholds: (**a**) aVAF < 50 and (**b**) aVAF > 100%. Time-to-event outcomes were analyzed and visualized using Cox proportional hazards methods. Abbreviation: aVAF, adjusted variant allele frequency.

Among patients with aVAF < 50%, the mOS was 31 months (95% CI = 24.3–37.7) compared with 29 months (95% CI = 26.2–31.9) among those with VAF ≥ 50%. After adjustment for pleural involvement, aVAF < 50% was not associated with OS (HR = 0.91; 95% CI = 0.41–2.02; *p* = 0.81), Figure 4a.

Patients with aVAF > 100% had a mOS of 43 months (95% CI = 25.1–60.9) compared with 29 months (95% CI = 24.7–33.3) among those with aVAF ≤ 100%. aVAF > 100% was not associated with OS (HR = 0.75; 95% CI = 0.33–1.73; *p* = 0.50), Figure 4b.

### 3.5. Relationship of Clinical Factors and Progression-Free Survival

The relationship between baseline clinicopathologic factors and PFS was evaluated using univariable analysis, as presented in Table 2. Higher ECOG performance status (≥2 vs. 0–1) and liver involvement were associated with inferior PFS, while *EGFR* exon 19 deletions (vs. L858R) were associated with superior PFS.

Variables significantly associated with PFS on univariable analysis were included in the multivariable model. ECOG performance status ≥2 (vs. 0–1; HR = 3.63; 95% CI = 1.89–6.96; *p* < 0.001), liver involvement (HR = 2.65, 95% CI = 1.35–5.22, *p* = 0.005), and *EGFR* exon 19 deletion (vs. L858R; HR = 0.50; 95% CI = 0.27–0.90; *p* = 0.021) were each independently associated with PFS.

## 4. Discussion

This study of patients with advanced *EGFR*-mt NSCLC treated with first-line osimertinib monotherapy demonstrated no significant association of *EGFR* VAF and either PFS or OS, even after adjustment for tumor cellularity. Although aVAF is often used by clinicians to discern clonal driver mutations from passenger mutations, our findings suggest that *EGFR* aVAF is not associated with clinical outcomes and has limited utility for risk stratification.

The lack of prognostic value associated with *EGFR* aVAF is likely multifactorial. Classical *EGFR* driver mutations generally occur early in the pathogenesis of *EGFR*-mt NSCLC [16]. As a result, the pretest probability of identifying a truly subclonal *EGFR* mutation—even in cases with low *EGFR* aVAF on tissue-based molecular testing—remains low. Instead, technical factors are likely the primary contributors of variability in *EGFR* aVAF, including differences in DNA quantity or quality, intratumor heterogeneity, bioinformatics pipelines, and PCR amplification bias [6,17]. In addition, tumor cellularity is visually estimated by pathologists, making it intrinsically subjective and potentially limited by interobserver variation [18]. In a comparable study of patients with *ALK* fusion-positive NSCLC treated with crizotinib, low *ALK* fusion aVAF was also not associated with PFS, suggesting that this observation may be reproducible across other oncogenic driver alterations [19].

Copy number variations (CNVs) are an additional factor that can influence VAF independent of clonality [17]. For example, amplification of the wild-type *EGFR* allele is anticipated to reduce *EGFR* aVAF by decreasing the proportion of mutant relative to wild-type alleles. Likewise, amplification of the mutant *EGFR* allele is expected to increase *EGFR* aVAF. Genomic instability is a hallmark of cancer, and CNVs are therefore relatively common [20]. Notably, amplification of the mutant *EGFR* allele has been described as a resistance mechanism to EGFR-targeted therapies by increasing the target-to-drug ratio and provided the rationale for exploring the high *EGFR* aVAF (>100%) category [21,22].

A recent study reported a negative association between PFS and an “extremely high” *EGFR* aVAF; however, only approximately 10% of patients met this predefined criterion [23]. In contrast, in our study, high aVAF—defined using a slightly lower exploratory threshold (>100%)—was not significantly associated with PFS or OS. These findings suggest that high aVAF may not be clinically meaningful in this context or that osimertinib retains sufficient potency to inhibit mutant *EGFR* signaling even when aVAF suggests a perturbed target-to-drug ratio. Correlation of aVAF with *EGFR* copy number status using fluorescence in situ hybridization or NGS platforms designed for copy number assessment was beyond the scope of this study and represents an area for future investigation.

This cohort is generally representative of patients with advanced *EGFR*-mt NSCLC. There was a higher proportion of females and patients with no or minimal tobacco exposure. In addition, *EGFR*-mt NSCLC has been reported to have high rates of brain metastasis, which was identified in more than 50% of patients in this cohort [24]. Importantly, we identified higher ECOG performance status, liver involvement, and *EGFR* L858R mutations as clinical factors independently associated with inferior PFS, consistent with prior reports [25,26]. In the era of multiple strategies to intensify front-line osimertinib therapy, such as combinations with amivantamab or platinum-based chemotherapy, risk stratification remains critical to optimize shared decision-making with patients [27].

As a retrospective analysis, this study may be limited by selection bias and confounding. To mitigate these effects, we assessed for confounding variables that were significantly different in *EGFR* aVAF subgroups and adjusted for them using a multivariable analysis. The only relevant variable was pleural involvement, which was not balanced between EGFR aVAF < 50% and ≥50%. The single-center design limits the generalizability of our findings to other institutions, especially those that use different approaches to *EGFR* mutation testing, such as real-time PCR rather than NGS. As in many real-world studies, imaging was performed at the discretion of the treating oncology team, which may introduce bias in PFS assessment; therefore, it should be interpreted with caution. For this reason, OS was evaluated as a secondary outcome and yielded similar results. Given the limited sample size, these findings warrant validation in larger cohorts.

Despite these limitations, this study has several strengths. It includes a well-characterized cohort of consecutively treated patients with advanced *EGFR*-mt NSCLC managed in a real-world setting. The findings are generally applicable to clinical practice, as VAF and tumor cellularity are routinely reported in molecular testing results. Finally, the study evaluates other relevant clinicopathologic prognostic factors in the context of the current standard-of-care EGFR-targeted therapy, osimertinib.

## 5. Conclusions

*EGFR* aVAF derived from tumor-based molecular testing was not associated with PFS or OS in patients with advanced *EGFR*-mt NSCLC treated with first-line osimertinib. In contrast, ECOG performance status, liver involvement, and *EGFR* exon 19 deletion mutations were associated with PFS, underscoring their potential value as clinically meaningful prognostic factors.

## Figures and Tables

**Table 1:** Baseline demographic and clinical characteristics of the study cohort stratified by adjusted *EGFR* variant allele frequency category.

Characteristic	Classification	Total (%)	aVAF < 50% (%)	aVAF > 100% (%)
Total	-	59	23	16
Age	Median (IQR)	70 (62–79)	73 (65–78)	63.5 (58.5–71)
Sex	Male	18 (30.5)	6 (26.1)	5 (31.3)
	Female	41 (69.5)	17 (73.9)	11 (68.8)
ECOG performance status	0–1	37 (62.7)	13 (56.5)	12 (75.0)
	>1	22 (37.3)	10 (43.5)	4 (25.0)
Smoking	None	30 (50.8)	13 (56.5)	5 (31.3)
	Light	3 (5.1)	2 (8.7)	0 (0)
	Current	2 (3.4)	0 (0)	1 (6.3)
	Former	24 (40.7)	8 (34.8)	10 (62.5)
PD-L1	0	18 (32.7)	8 (38.1)	3 (18.8)
	1–49	25 (45.5)	9 (42.9)	8 (50.0)
	≥50	12 (21.8)	4 (19.0)	5 (31.3)
*EGFR* mutation	L858R	30 (50.8)	11 (47.8)	8 (50.0)
	Exon 19 deletion	29 (49.2)	12 (52.2)	8 (50.0)
Sites of involvement	Brain	33 (55.9)	10 (43.5)	11 (68.8)
	Bone	29 (49.2)	9 (39.1)	8 (50.0)
	Pleura	23 (39.0)	13 (56.5)	4 (25.0)
	Liver	16 (27.1)	5 (21.7)	4 (25.0)
	Adrenal	12 (20.3)	6 (26.1)	4 (25.0)

Abbreviation: IQR, interquartile range; aVAF adjusted variant allele frequency; PD-L1 programmed death ligand 1.

**Table 2:** Association between baseline clinical factors and progression-free survival using a univariable proportional Cox regression model.

Variable	Hazard Ratio	95% CI	*p*-Value
Age (continuous)	1.01	0.99–1.04	0.31
Sex (female vs. male)	1.32	0.69–2.51	0.41
ECOG performance status (≥2 vs. 0–1)	2.58	1.42–4.71	0.002
Smoking history (any vs. none)	0.99	0.56–1.77	0.99
EGFR (exon 19 vs. L858R)	0.52	0.288–0.936	0.029
Brain metastasis	1.26	0.71–2.26	0.43
Liver metastasis	1.97	1.05–3.70	0.034
Pleural metastasis	1.05	0.58–1.89	0.87
Adrenal metastasis	1.44	0.71–2.93	0.31
Bone metastasis	1.67	0.93–2.99	0.088
Adjusted VAF (continuous)	1.00	0.99–1.01	0.97

Abbreviations: ECOG, Eastern Cooperative Oncology Group; 95% CI, 95% confidence interval; VAF, variant allele frequency.

## Data Availability

Data are not publicly available due to patient privacy and institutional restrictions but are available from the corresponding author upon reasonable request, subject to institutional and ethics approval.

## Supplementary Materials

The following supporting information can be downloaded at: https://www.mdpi.com/article/10.3390/curroncol33080439/s1, Table S1: The relationship of baseline characteristics with EGFR aVAF categories.
