# Supplementary material for: The Prognostic Significance of EGFR Adjusted Variant Allele Frequency on First-Line Osimertinib Efficacy in Advanced EGFR-Mutant NSCLC
Source: Curr Oncol. 2026 Jul 23;33(8):439. doi: 10.3390/curroncol33080439 (PMC13511178; doi:10.3390/curroncol33080439)
Supplement: Supplementary file 1 [file curroncol-33-00439-s001.zip › curroncol-4410194-supplementary.pdf]

SUPPLEMENTAL INFORMATION

Supplementary Table 1: The relationship of baseline characteristics with *EGFR* aVAF categories.

Variable	Description	aVAF <50%	aVAF ≥50%	p-value	aVAF ≤100%	aVAF >100%	p-value
Age	Median (IQR)	73 (65-78)	66 (61.5-80)	0.32	73 (62-81)	63.5 (58.5-71)	0.59
Sex	Male	6 (26.1)	12 (33.3)	0.56	13 (30.2)	5 (31.3)	0.94
	Female	17 (73.9)	24 (66.7)		30 (69.8)	11 (68.8)	
ECOG PS	0-1	13 (56.5)	24 (66.7)	0.43	25 (58.1)	12 (75.0)	0.23
	>1	10 (43.5)	12 (33.3)		18 (41.9)	4 (25.0)	
Smoking history	Any	13 (56.5)	19 (52.8)	0.49	18 (41.9)	11 (68.8)	0.07
	None	10 (43.5)	17 (47.2)		25 (58.1)	5 (31.3)	
PD-L1	0%	8 (38.1)	10 (29.4)	0.79	15 (28.5)	3 (18.8)	0.31
	1-49%	9 (42.9)	16 (47.1)		17 (43.6)	8 (50.0)	
	≥50%	4 (19.0)	8 (23.5)		7 (17.9)	5 (31.3)	
EGFR mutation type	Exon 19 deletion	12 (52.2)	17 (47.2)	0.71	21 (48.8)	8 (50.0)	0.94
	L858R	11 (47.8)	19 (52.8)		22 (51.2)	8 (50.0)	
Brain metastasis	Present	10 (43.5)	23 (63.9)	0.12	22 (51.2)	11 (68.8)	0.23
Pleural metastasis	Present	13 (56.5)	10 (27.8)	0.027	19 (44.2)	4 (25.0)	0.18
Liver metastasis	Present	5 (21.7)	11 (30.6)	0.46	12 (27.9)	4 (25.0)	0.82
Adrenal metastasis	Present	6 (26.1)	6 (16.7)	0.38	8 (18.6)	4 (25.0)	0.59
Bone metastasis	Present	9 (39.1)	20 (55.6)	0.22	21 (48.8)	8 (50.)	0.94

Abbreviations, aVAF adjusted variant allele frequency; ECOG PS Eastern Cooperative Oncology Group performance status; PD-L1, programmed death ligand 1; IQR, interquartile range.
